# Parent mediated intervention programmes for children and adolescents with neurodevelopmental disorders in South Asia: A systematic review

**DOI:** 10.1371/journal.pone.0247432

**Published:** 2021-03-11

**Authors:** Kamrun Nahar Koly, Susanne P. Martin-Herz, Md. Saimul Islam, Nusrat Sharmin, Hannah Blencowe, Aliya Naheed

**Affiliations:** 1 Initiative for Non-Communicable Diseases, Health Systems and Population Studies Division, International Centre for Diarrhoeal Disease Research, Bangladesh, Dhaka, Bangladesh; 2 Department of Pediatrics, Division of Developmental Medicine, University of California San Francisco, San Francisco, CA, United States of America; 3 Department of Psychology, Bangabandhu Sheikh Mujibur Rahman Science and Technology University, Dhaka, Bangladesh; 4 Maternal Adolescent Reproductive and Child Health Centre, London School of Hygiene and Tropical Medicine, London, United Kingdom; Birkbeck University of London, UNITED KINGDOM

## Abstract

**Objective:**

Parent-mediated programmes have been found to be cost effective for addressing the needs of the children and adolescents with Neurodevelopmental Disorders (NDD) in high-income countries. We explored the impact of parent-mediated intervention programmes in South Asia, where the burden of NDD is high.

**Methods:**

A systematic review was conducted using the following databases; PUBMED, MEDLINE, PsycINFO, Google Scholar and Web of Science. Predefined MeSH terms were used, and articles were included if published prior to January 2020. Two independent researchers screened the articles and reviewed data.

**Outcomes measures:**

The review included studies that targeted children and adolescents between 1 and 18 years of age diagnosed with any of four specific NDDs that are commonly reported in South Asia; Autism Spectrum Disorder (ASD), Intellectual Disability (ID), Attention Deficit Hyperactivity Disorder (ADHD) and Cerebral Palsy (CP). Studies that reported on parent or child outcomes, parent-child interaction, parent knowledge of NDDs, or child activities of daily living were included for full text review.

**Results:**

A total of 1585 research articles were retrieved and 23 studies met inclusion criteria, including 9 Randomized Controlled Trials and 14 pre-post intervention studies. Of these, seventeen studies reported effectiveness, and six studies reported feasibility and acceptability of the parent-mediated interventions. Three studies demonstrated improved parent-child interaction, three studies demonstrated improved child communication initiations, five studies reported improved social and communication skills in children, four studies demonstrated improved parental knowledge about how to teach their children, and four studies reported improved motor and cognitive skills, social skills, language development, learning ability, or academic performance in children.

**Conclusion:**

This systematic review of 23 studies demonstrated improvements in parent and child skills following parent-mediated intervention in South Asia. Additional evaluations of locally customized parent-mediated programmes are needed to support development of feasible interventions for South Asian countries.

## Introduction

According to the Diagnostic and Statistical Manual of Mental Disorders, 5th Edition (DSM-5), neurodevelopment disorders (NDDs) are a group of conditions, with onset in the developmental period, typically characterized by developmental delays that produce impairments in personal, physical, social, academic, or occupational functioning. Autism Spectrum Disorder (ASD), Intellectual Disability (ID; updated from the former diagnosis of mental retardation in 2013), Attention Deficit Hyperactivity Disorder (ADHD) and Cerebral Palsy (CP) are the most common NDDs in children, and all have impacts on child health, well-being and educational attainment [1, 2]. All these conditions share some common characteristics, such as delays in functional or communication skills or both, and cause problems in physical, learning and behavioural functioning [2, 3]. ASD additionally may have signs of social and behavioural differences, leading to higher rates of parent stress and potential for documented mental health conditions [4–7].

The majority of children with NDDs live in South Asia, albeit epidemiological data on NDDs are scarce from the region [1]. In low and middle income countries (LMICs) in South Asia, 7.6 per 1000 children have at least one of the four common NDDs, and the prevalence of an individual NDD varies widely across South Asian countries [1, 8]. According to a systematic review, the prevalence of ASD ranges from 0.09 to 1.07 percent in South Asia, and this burden accounts for approximately 9% of the Disability-Adjusted Life Years (DALYs) for this region [9]. A meta-analysis including literature from South Asian countries between 1987 and 2007 estimated that the rate of ID ranged from 0.93 to 156.03 per 1000 children, with the lowest rate reported in India and the highest rate reported in Bangladesh [10–12]. While the global prevalence of ADHD has been estimated to be 7.2 percent [13], a limited indexed literature from South Asian countries suggests that ADHD prevalence ranges between 5% in India and 18% in Pakistan [14–17]. The incidence of CP is reported to be 2 to 3 per 1000 live births globally, with rates 5 to 10 times higher reported in resource poor countries, including South Asia [18–22]. Overall, these data suggest a high burden of NDDs in South Asia and highlight a compelling need for exploring interventions that can effectively support children with NDDs in the region.

Evidence suggests that comprehensive packages of interventions and therapies can improve outcomes and maximize functional abilities [23–27]. Intervention packages may include applied behaviour analysis (ABA), educational therapies following structured teaching plans, social skill training, physical therapy, occupational therapy or speech-language therapy, with the choice of therapy based on the individual’s NDD type, severity, and functional needs [25, 27–30]. A few studies have demonstrated that parents can play a critical role in implementing therapeutic programmes [31–34]. While parental involvement in intervention programmes may differ across NDDs, it can result in improvements in several areas, including maladaptive behaviours, social communication, speech-language development, physical and emotional health, and motor functioning [35–53]. It can also positively impact quality of life by increasing effective parenting skills and parent understanding of their child’s NDD, and reducing parent stress [33, 54, 55]. More specifically, a number of studies have demonstrated that parental stress can be reduced and the whole family environment can be improved by increasing parental skills to improve their child’s condition [45, 56, 57]. Parent-mediated programmes have been found to be cost effective in high income countries (HIC) and are recommended as a means of sustainable support for children with NDDs [56–63]. In LMICs including South Asia, opportunities for professional therapies for children with NDDs are limited by a dearth of available services, particularly in hard to reach areas, and a scarcity of appropriately trained health care professionals. Further, health systems in South Asia are not well equipped to deliver care that supports parents of children with NDD, hence parent-mediated programs may be a pragmatic strategy for supporting children and adolescents with NDD in South Asia [31, 64].

In high-income countries, parent-mediated programmes are generally embedded in regular intervention programs administered through special educational centres [57, 59, 65], while most children with NDDs in low-income countries do not have access to professional or educational support due to an inadequate number of specialized clinics and schools and a shortage of qualified special educators [66, 67]. Training programs to improve parents’ skills could create an alternative workforce in a setting where institutional support for children with NDD is limited, and this could be a sustainable strategy to address the needs of a large number of children with NDDs, particularly in the low-resource settings in South Asia [31, 64, 68]. We conducted a systematic review of the literature to understand if parent-mediated intervention programmes are acceptable by parents of children and adolescents with NDDs in South Asia. We defined a parent-mediated intervention as acceptable if it was associated with a positive change in the performance of parents of children and adolescents with NDD, or the children and adolescents with NDD or both in terms of attitude, knowledge, and management skills in the parents, and developmental or behavioural symptoms in the child and adolescent.

## Materials and methodology

This review broadly considered parent-mediated intervention programmes for children with one of four different NDDs (ASD, ID, ADHD and CP) from one of the South Asian countries including Afghanistan, Bangladesh, Bhutan, India, Maldives, Nepal, Pakistan, and Sri Lanka. It further documented changes in parent and child behaviour or skills as a direct or indirect effect of the intervention.

### Inclusion criteria

This review included studies that (1) targeted parents of children and adolescents from 1 to 18 years of age; (2) with a diagnosis of ASD, ID, ADHD or CP according to one of the following diagnostic criteria: DSM-IV, DSM-5, the International Statistical Classification of Diseases and Related Health Problems, tenth revision (ICD-10); (3) conducted research in one or more of the South Asian countries; (4) was published in any language in an indexed international scientific journal prior to January 2020; (5) utilized an experimental study design, including either a Randomized Control Trial (RCT), quasi-randomized trial or pre-post intervention study design with outcomes assessed at baseline and end line; (6) reported a child outcome and/or a parent outcome; and (7) involved parents directly as trainers with their children or adolescents in order to manage difficulties related to one of the outlined NDDs.

#### Population

Selected studies needed to include parents of children and adolescents aged 1 to 18 years diagnosed with at least one of the four selected NDDs: ASD, ID, ADHD or CP as defined above.

#### Intervention

This systematic review included intervention studies conducted through individual or group sessions in one or more South Asian countries that involved parents as implementers. This review has also included studies that adapted intervention programmes developed in high-income countries and applied them in a South Asian country [23].

#### Outcomes

The systematic review included studies that reported on changes either in parent outcomes, child outcomes or both. Parent outcomes included improvement in parent-child interaction, parental social and communication skills, and parental knowledge of NDDs. Child outcomes included improvement of motor, cognitive, social or language development, reductions in violent or self-injurious behaviour or temper tantrums, or improvements in activities of daily living, learning ability, impulse control, academic performance or social withdrawal.

#### Search strategy

We followed the PRISMA Guidelines of reporting [69]. The following databases were searched comprehensively for relevant articles: PubMed, MEDLINE, PsycINFO, Web of Science and Google Scholar. The search terms for identifying relevant articles is described in the **S3 Table**.

Initial search terms included “Autism” OR “Autistic” OR “Autism Spectrum Disorders,” “Intellectual Disability” OR “Mental Retardation,” and “Parental Training” OR “Parent mediated programmes,” OR “Parental intervention programmes” and the name of each South Asian country separated by “OR”. A secondary search included “Attention Deficit Hyperactive Disorder” OR “Cerebral Palsy” OR “Parent Training” OR “Parent mediated programmes,” OR “Parental intervention programmes” with the name of each South Asian country separated by “OR.” Additional articles were identified using the reference lists from the retrieved articles and the Google Scholar database.

Two reviewers (KNK and NS) independently searched the literature and cross-checked selected studies to ensure the quality and accuracy of the information using a priori prioritization and sequential exclusion techniques. A third reviewer (MSI) resolved any disagreements raised between the first two reviewers (KNK and NS). Overall, disagreements were found in 2.4% of articles (30/1231). The value of kappa statistics was 0.84 indicating strong agreement between the two reviewers. Duplicate articles were removed.

#### Quality assessment

Study quality was assessed according to standard quality assessment criteria described by Kmet [70, 71]. The Kmet appraisal checklist uses 14 items, each with a 3-point ordinal system (i.e., yes, partial, and no) to assess the methodological quality of a research paper [70, 71]. Two reviewers independently reviewed each individual study regarding study objectives, methodology, study design, eligibility criteria, appropriate sample size and power, description of experimental and control groups, random allocation of participants, blinding procedures, descriptions of the analytic methods, estimation of outcome variance, methods for controlling potential confounder(s), follow-up length, retention rate, generalizability and acceptability of the findings. Fourteen areas were scored based on the degree to which the specific criteria were met (“yes” = 2, “partial” = 1 and “no” = 0). A summary score was calculated for each study by summing area scores and dividing by the total possible score. Scores were categorized into four quality levels prior to selection for final analysis: >80% = strong, 70–80% = good, 50–69% = adequate, and <50% = poor. **S1 Table** includes the overall methodological quality scores and the quality of evidence that is identified by following the Kmet guidelines (**S1 Table**).

#### Data management and analysis

Title and abstract screenings were completed independently by two reviewers (KNK and MSI). Any discrepancies were resolved by a 3rd reviewer (NS). Full text screenings of eligible manuscripts were completed by KNK and NS and information collected in a data extraction sheet in Microsoft Excel (version 2013). Characteristics of interest included: (1) study title, (2) year of publication, (3) country where the study was conducted, (4) objectives of the study, (5) sample description, (6) type of intervention and (7) key findings, including effect size of outcomes. Information from all eligible studies was synthesized for each NDD separately and a narrative synthesis was used to summarize findings for each NDD.

## Results

**Fig 1:**
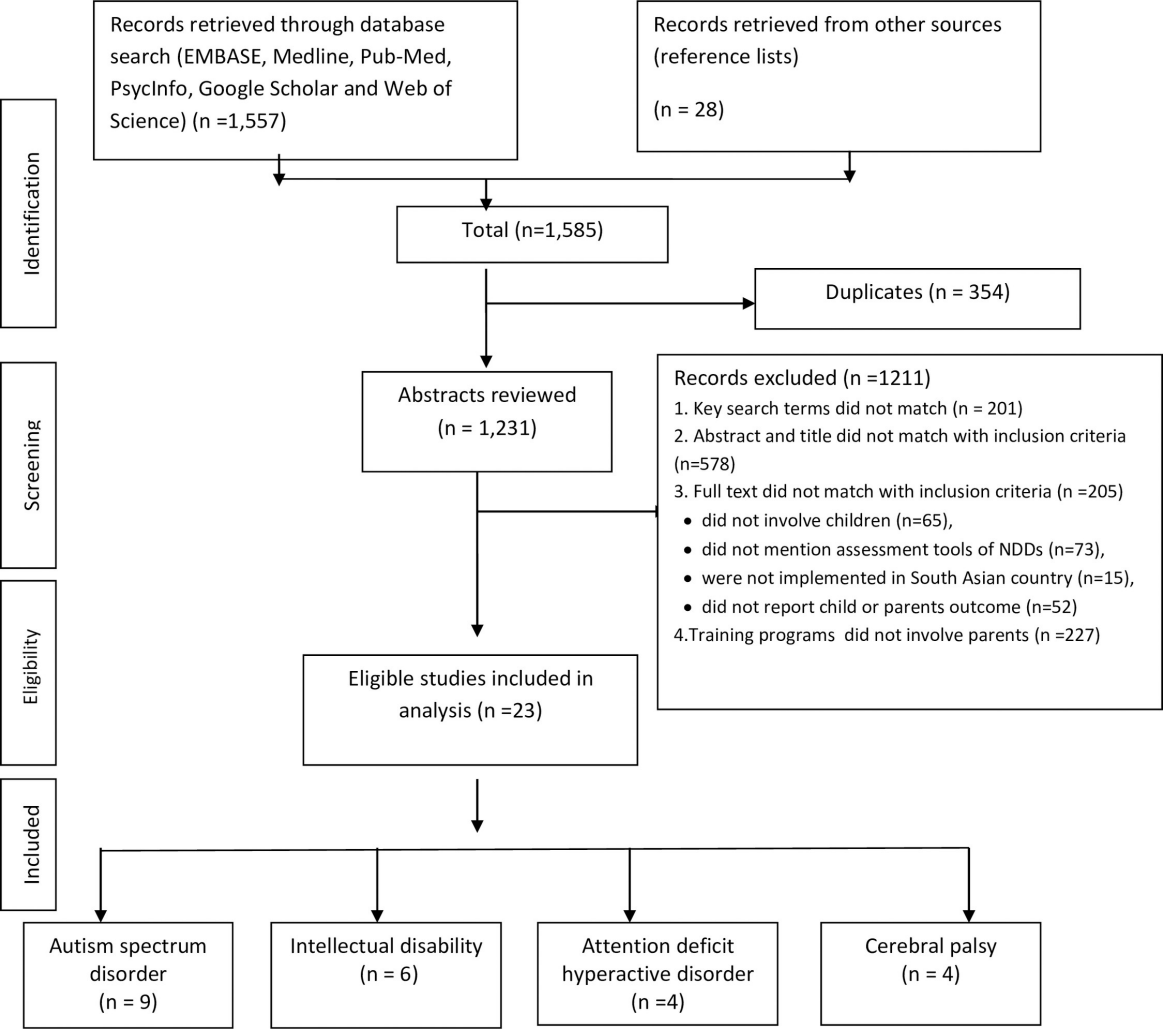
A flow diagram of study selection for the systematic review.

Database search identified a total of 1557 articles all published in English language, and 28 additional abstracts were retrieved by searching other sources manually published in English. From a total of 1585 published articles, 354 duplicate articles were removed, leaving 1231 abstracts for review, and all of them were published in English. Following screening of the abstracts, 1211 articles were excluded for the following reasons: the key term search did not match (n = 201), title and abstract did not match inclusion criteria (n = 578), full text did not match with inclusion criteria (n = 205), and training programs did not involve parents (n = 227). Finally, a total of 23 eligible studies met the inclusion criteria for full text review and were included in the final review (**Fig 1**).

We initially analyzed information related to each of the four NDDs separately, considering each NDD a theme, and laid out results as subthemes under each theme. We divided the results into eight subthemes: (i) study characteristics, (ii) tools used, (iii) methodology, (iv) quality, (v) adaptation process of the intervention programmes, (vi) components of the interventions, (vii) modality of the interventions, and (viii) changes in the outcome.

### Autism Spectrum Disorder (ASD)

#### (i) Study characteristics

Nine studies conducted between January 2010 and January 2020 among parents of children or adolescents with ASD were included in the final analyses. Eight studies were implemented in India, and one study was collaboratively implemented in both India and Pakistan.

#### (ii) Tools used

For participant inclusion, only three of nine studies used a formal diagnostic tool, with two using the INCLEN Diagnostic Tool for Autism (Juneja et al., 2014) and one used the ADOS tool developed by C Lord et al. in 1989 [72–74]. None of these studies described adaptation or validation of these tools for the study context. The other six studies used DSM (4) or ICD-10 (1) or clinical (1) criteria implemented by clinicians. Assessment tools included ASD screening tests/checklists, ASD observation scales, and assessments of adaptive functioning, social maturity, speech-language skills, and general development of young children. The Vineland Social Maturity Scale (VSMS) is an international tool that was developed by an American psychologist Edgar Arnold Doll in 1986. It is an assessment tool that measures the adaptive skills of the child in eight areas. No information was provided as to whether the studies used a language/culture adapted version, or the original version developed in English. This scale is used to assess children or adolescents aged 0–16 years in the areas of self-help general, self-help dressing, self-help eating, self- direction, locomotion, communication, occupation and socialization (**Table 1**).

**Table 1:** Characteristics of the included studies for children and adolescents with Autism Spectrum Disorder (ASD).

Author, year of publication	The process of adaptation	Description of participants	Tools for assessment	Service providers	Components of the intervention	Duration of each treatment/ intervention	Modality of the training process	Times of evaluation
**Krishnan et al. (2016)**	N/A	Parents and children with ASD Dropout Rate: N/A	Psycho-Educational Profile-Revised (PEP-R); Childhood Autism Rating Scale (CARS); Gesell Developmental Schedules(GDS); Vineland Social Maturity Scale (VSMS)	Occupational therapist	Clinic-based, multi-component early intervention package with 3 components: 1. Standard intervention protocol that included training in self-care skills, social skills and control of problem behaviour using special education and behavioural techniques 2. Parent education about ASD, using an interactive group psycho-educational technique, the Psycho-Educational Profile-Revised (PEP-R) intervention, which included teaching activities and treatment for children with ASD and developmentally disability, teaching strategies for parents and for individual assessment 3. The Carolina Curriculum for Infant and Toddlers with Special Needs (CCITSN) module, which used a developmental approach to address ASD symptoms with focus on cognitive and motor skills, communication and social adaptation.	12 weeks	• Child was engaged in play routines and social stories • Each parent–child dyad received applied behaviour analysis and parent interactive skills training using principles of rewarding and guided practice • Intervention was in closed group sessions.	After 12 weeks of intervention
**Rahman et al. (2016)**	• A UK team trained and supervised local specialists who then trained and supervised the local staff (implementation specialists) and continued to provide online support as necessary during the trial. • Separate local experts at each site trained and supervised the local assessors and the health workers • All written information and questionnaire instruments were translated	Parents and children with ASD Dropout Rate: 9.23%	Modified Checklist for Autism in Toddlers (M-CHAT); Vineland Adaptive Behaviour Scales (VABS)	Local specialist trained the local non-specialist health worker	• A naturalistic approach for scaffolding and developing communication skills • Parent-mediated Intervention for Autism Spectrum Disorder in south Asia (PASS) • In the experimental group, PASS was delivered individually.	6 months	• One-to-one clinic or home sessions between health worker and parent with the child present • Health workers were supported with semi-structured scripts. • One hour sessions every 2 weeks for 6 months (12 sessions) • Initial home visit from non-specialist health worker, supported by a local specialist, explored parents’ beliefs about the nature and origin of ASD and other factors that might affect engagement, including individual learning styles of the target parent • Each parent-child session was videotaped and reviewed in detail with parents for progress since last session, fidelity to treatment goals and planning next steps. • Parents were asked to spend 30 minutes a day between clinic sessions practicing predefined strategies at home.	After 6 months of intervention
**Brezis et al. (2015)**	• Culturally adapted Western ASD-training methods (e.g., TEACCH, Applied Behavioural Analysis, Floor time) • No information about the adaptation process provided	Parents of children with ASD Dropout Rate: 5%	Five Minute Speech Sample (FMSS)—Parents described their child in terms of behaviours, primarily social and cognitive skills	Trainer	• Incorporated Western ASD-training methods (e.g., TEACCH, Applied Behavioural Analysis, Floor time) after cultural adaptations	3 months	Training included daily group and one-on-one activities with the children (led by the parents) and group discussions for the parents.	After 3 months of intervention
**Louis and Kumar (2015)**	N/A	Fathers of children with ASD Dropout Rate: N/A	Play Based Observation (PBO); Griffiths Mental Development Scales-Extended Revised (GMDS-ER); Vineland Social Maturity Scale (VSMS)	Psychologist and Therapist	Fathers were taught • to use a list of words during play and to reinforce responses of attachment and reciprocity. • simple messages to help the child perform activities of daily living	3 months	• Fathers in the treatment group attended a clinic-based program, consisting of three 1-hour sessions • In the first session, fathers observed how the therapist interacted with the child, and engaged the child one-to-one, then demonstrated how they would engage the child at home.	6 months after training
**Nair et al. (2014)**	N/A	Parents of children with ASD Dropout Rate: N/A	CARS; VSMS; Receptive Expressive Emergent Language Scale	Therapist	• Parents trained to identify developmental age of child, using an intervention kit including inexpensive materials available at home or the local market • Parents educated in simple behavioural strategies (e.g., prompt and rewards) to support gains in language and socialization and reduce repetitive behaviours • Cognitive, academic, and prevocational skills were also addressed. • Parents encouraged to give intervention daily at home • At each follow-up visit, improvements were noted and recorded as “emerging” or “attained.” • Parents were also advised to place the child in a playschool to improve the group-based stimulation of various target symptom clusters.	3 months	• Each clinical session took about 15–20 minutes. • Intervention strategies were demonstrated to parents, and they were advised to practice at home. • A speech therapist gave parents 15 to 20-minute one-on-one sessions and targeted one or two language skills per visit for teaching at home.	After 3 months
**Juneja et al. (2012)**	Initial comorbidity screening questionnaire for parents was adapted from work done in the United Kingdom	Parents and children with ASD Dropout Rate: 55.55%	CARS; Autism Behaviour Checklist (ABC); Early Developmental Profile (EDP); VSMS; Receptive Expressive Emergent Language Scale	Pediatricians, Clinical Psychologists, Speech Therapists, Special Educators, and Occupational Therapists	• Individualized program designed to improve child attention, communication, social skills and behaviour • Principles of the Naturalistic method/Milieu method used to create situations during the daily activities of the family to develop joint attention behaviours, such as index pointing, gaze switching, showing, and holding out objects • Play-based activities to develop joint attention behaviours, social skills, and communication included ball play, toy cars, clay, colored stones, balloons, water play, painting and coloring Principles of Lovaas’ Applied Behaviour Analysis used to improve communication with gestures and words	6 months	• A specialist demonstrated methods to parents • Parents acted as therapist • Parents expected to spend 45–90 minutes daily in one-to-one setting with child	6 months after intervention
**Divan et al. (2019)**	• Senior clinicians conducted one workshop with parents. • Researchers independently plotted intervention strategies from source materials within the intervention framework.	Parents and children with ASD Dropout Rate: 15%	Brief Observation of Social Change (BOSCC); Dyadic Communication Measure for Autism (DCMA); Developmental Behaviour Checklist (DBC); Standard Vineland Behaviour Scale (VABS); PHQ9; Measure adapted by Research on Autism and Families in India (RAFIN)	Health worker, under supervision of a specialist	• A naturalistic approach for scaffolding and developing communication skills • Used parent-mediated Intervention for Autism Spectrum Disorder in south Asia (PASS) • In the intervention group, PASS Plus was implemented along with treatment as usual.	6 months	• One-on-one home-based sessions • Video feedback • The PASS facilitator visits the home of the child, gives the parent a standard box of toys and requests them to play with their child. • The novel Plus modules are delivered using a manualized clinical decision algorithmic approach address common comorbidities using a psychosocial approach. • Home practice	After 6 months of intervention
**Padmanabha et al. (2019)**	N/A	Parents and children with ASD Dropout Rate: N/A	Parent Rated 10-Item Likert Scale (PRILS-10); Children’s Global Assessment Scale (CGAS); Pediatric Quality of Life Inventory 4.0 (Peds™); Childhood Autism Rating Scale (CARS); Vineland Social Maturity Scale (VSMS) Intelligent Quotient (IQ)	An expert committee including pediatric neurologist, child psychologist; and occupational therapist designed the intervention activities.	• Standard Therapy (ST) group: Intervention included only the institution-based standard care for ASD • Sensory Intervention (SI) group: Intervention included predesigned structured Home-Based Sensory Interventions (HSBI) and also Standard Therapy • Activities of HSBI included tactile activities, vestibular visual stimulation, and auditory stimulation	12 weeks	• Videotaped • Home-based sensory interventions (HBSI)Children in the SI group • Received pre-designed structured HBSI. An expert committee comprised of pediatric neurologists, child psychologist and occupational therapist designed the activities • The parents/caregivers were given hands on training on HBSI, along with a manual and training videos by the principle investigator	During the intervention at 2, 4, 8 and 12 weeks
**Manohar et al. (2019)**	The components of the study intervention were adapted from intervention practices followed in specialist child psychiatry centers and institutes of national importance in India, which include the principles of Naturalistic Developmental Behavioural Interventions (NDBI).	Parents and children with ASD Dropout Rate: 6%	Childhood Autism Rating Scale (CARS); Vineland and Social Maturity Scale; Family interview for stress and coping; Point Visual Analog Scale	Pediatrician, Psychiatrist, Speech and Occupational Therapist	**Intervention group:** • Outpatient-based intervention included 4 components: joint attention, verbal and motor imitation, social engagement and adaptive skill training. Components based on Naturistic Developmental Behavioural Interventions (NDBI), which involve developmentally appropriate prerequisite skills in a naturalistic environment using family friendly behavioural strategies, and a strong emphasis on home-based parent mediated intervention • Visit 1 and 2: Pre-intervention assessment, educating parents and addressing stress from a cultural perspective • Visit 1–3: Intervention • Visit 3–5: Follow-up, review of intervention and ongoing support • This group also received the treatment as usual (TAU). **Active Control Group:** • TAU • Setting was involved in institutional treatment for ASD	12 weeks	• Delivered on a one–to-one basis over five sessions • Monthly visits to the treating doctor • Referral for speech and language interventions and occupational therapy are initiated and pharmacological intervention for comorbidities are considered on a case–by-case basis.	Endline survey conducted since baseline to 12 week follow up

#### (iii) Methodologies

Among the nine studies, five were RCTs [23, 32, 33, 52, 65] and four were pre-post intervention studies [31, 75–77]. The RCTs implemented locally customized intervention programmes, including a multi-component early intervention package, interactive group psychoeducation and a naturalistic approach for scaffolding and developing communication skills to train parents about various aspects of ASD. The naturalistic approach was based on the Naturalistic Developmental Behavioral Interventions (NDBI), which involve the use of natural contingencies and behavior strategies in natural settings with some sharing of control between the child and therapist, and targeted skills areas that are commonly challenging for children on the autism spectrum [52, 78]. The pre-post studies integrated multiple intervention components that addressed adaptive behaviour and behaviour problems (**Table 1**).

#### (iv) Quality

By Kmet criteria, six studies were of strong quality, one was good, and two studies were of adequate quality (**S1 Table**). The two RCTs clearly described the process of recruiting participants and drop out or refusal to participate. One of the RCTs was conducted in two different countries [23]. The four pre-post intervention studies all had methodological concerns. All had a small sample sizes and did not include a control arm to negate potential confounding factors. One pre-post study was limited by a retrospective study design [75] (**Table 1**)

#### (v) Adaptation process of the intervention programmes

Four studies adapted their interventions from the other countries. Rahman et al. (2016) and Divan et al. (2019) adapted intervention programmes from the United Kingdom that were later translated and culturally adapted [23, 65]. Another study conducted in South India applied a brief parent-mediated intervention adapted from good intervention practices utilized in specialist child psychiatry centre in India that had been primarily adapted from USA-based programmes. These included the principles of Naturalistic Developmental Behavioural Interventions (NDBI) and family friendly behavioural strategies [32]. A fourth study, which was conducted in India, incorporated Western ASD-training methods (e.g., TEACCH, Applied Behavioural Analysis, Floor time) with cultural adaptations, but there was no detailed information about the adaptation process [27] (**Table 1**).

#### (vi) Components of the interventions

**Parent components:** Several intervention programmes were implemented in these studies. Rahman et al. (2016) customized parent-mediated interventions for ASD in South Asia (such as PASS, which shares the core theoretical foundations of the original Pre-school Autism Communication Therapy, PACT), while in another study western ASD-training methods were incorporated with the necessary cultural adaptations [23, 31]. In 2019, an extended study of the original PASS was presented called PASS Plus. This included new modules to address co-morbidity in a more demanding setting. Lay health workers were trained to support parents in implementing this intervention [65]. One of the studies utilized clinic-based, multi-component early intervention packages to teach parents about ASD. Intervention components included standard intervention protocols, the Psycho-Education Profile intervention (PEP-R), and the Carolina Curriculum for Infants and Toddlers with Special Needs (CCITSN) [75] (Table 1 and S2.1 Table in S2 Table). Out of nine studies on ASD only two studies assessed impact of parent mediated training programs on the parental knowledge.

**Child and adolescents components:** One individualized programme was designed to improve the attention, communication, social skills, and behavior of children or adolescents. Juneja et al. (2012) used principles of the Naturalistic/Milieu method to create situations during family daily activities that supported development of joint attention behaviours, such as index finger pointing, gaze switching, showing, and holding out objects [77]. A similar study by Manohar et al. (2019) also involved Naturalistic Developmental Behavioural Interventions (NDBI) to teach developmentally appropriate and prerequisite skills in a naturalistic environment using family friendly behavioural strategies [52] (**Table 1**).

#### (vii) Modality of the interventions

**Settings:** Eight studies were conducted in clinic/hospital settings, and one was carried out in a training centre. **Duration:** The duration of intervention varied from three to six months. **Service providers:** Five studies reported that the interventions were delivered by therapists and the other studies reported that various providers other than therapists delivered the intervention, including pediatricians (2 studies), special educators (1 study), a trainer (1 study), a psychiatrist (1 study), a psychologist (1 study), and health workers (1 study). **Ways of delivering:** The intervention programmes were delivered by closed-group sessions [32], one-on-one sessions [31], and home-based sessions [23, 33, 52, 65, 75–77]. **Time to evaluation or end-line:** Duration of follow up varied from 3 months to 6 months after intervention. **Dropout rate:** Among 9 studies, 5 mentioned the dropout rate of participants, with a range between 5% and 56% of participants not completing the program [23, 31, 52, 65, 77] (**Table 1**).

#### (viii) Changes in the outcome

**Parent outcome:** All studies except one (Juneja et al., 2012) mentioned a parent outcome [77]. Two studies found that parent-mediated interventions had a positive impact on parent-child interactions and parental synchronous interactions, improved the adaptive behaviour of children, and increased fathers’ involvement in the intervention process [23, 68]. The remaining two RCTs showed improvement in parental depressive symptoms, reduced parent distress and improved family coping [52, 65]. One pre–post intervention study found that parents were less likely to compare their children to other children after the intervention, suggesting increased acceptance of their child’s diagnosis [31]. However, the programme was implemented among families with higher income, and the parents were highly educated [31], factors which may cause a bias and reduce generalizability of findings [79, 80]. Reassuringly, a separate study additionally demonstrated changes in the efficacy of parents who were illiterate and belonged to low socioeconomic classes [68] (**Table 1**).

**Child and adolescent outcome:** All nine studies highlighted at least one child. Two pre-post studies and one RCT suggested improvements in perception, fine and gross motor skills, eye-hand coordination, cognitive performance and/or verbal skills among children with mild to moderate and severe ASD [33, 75, 76]. One of these pre-post intervention studies showed strong effects on adaptive behaviour and behavioural problems as well [75]. Three studies suggested significant improvements in general development, social skills, expressive language, adaptive behaviour including socialization, social age, self-help dressing and eating, as well as improvement in behaviour and reduction in symptoms on the Childhood Autism Rating Scale [76, 77, 81]. Two studies reported improvement in communication initiations with parents and in child play skills [23, 32] (**Table 1**).

### Intellectual disability (ID)

#### (i) Study characteristics

Six studies were conducted among parents of children or adolescents with ID. Five were carried out in India and one in Pakistan.

#### (ii) Tools used

For participant inclusion, one study used the Behavioral Assessment Scale for Indian Children with Mental Retardation (BASIC-MR), which was developed in the studies’ regional context and then locally customized; two studies used the BINET-KAMAL scale of intelligence, a validated Indian adaptation of the Kamath’s Test of Intelligence; one study used DSM-IV criteria and one did not provide information on diagnosis. Tools included assessments of behaviour, general development, intelligence, adaptive functioning, and social maturity, as well as evaluations of parent attitudes towards their children with ID and goal attainment. Two studies in India used a tool solely developed in the Indian context to assess behaviour change among children, the BASIC-MR (Behavioural Assessment Scale for Indian Children with Mental Retardation) and PAM-ID (Parental Attitude Scale, Bhatti et al. 1985) [51, 81, 82] (**Table 2**).

**Table 2:** Characteristics of the included studies for children and adolescents with Intellectual Disability (ID).

Author, year of publication	The process of adaptation	Description of participants	Tools for assessment	Service providers	Components of the intervention	Duration of the treatment/intervention	Modality of the training process	Time of evaluation
**Kurani et al. (2009)**	The assessment scale for children has been adapted from the Portage Parent Program by Richard D. Boyd and Susan M. Bluma (1977), USA, which has been widely used in India since 1980. Translated into Hindi in 1987, thus making it an easily accessible	Children with ID and their parents Dropout Rate: N/A	Binet–Kamath Scale of Intelligence; Global Assessment Scale (GAS); Malin’s Intelligence Scale for Indian Children; VSMS	Therapists and teachers	• Therapeutic intervention (1:1) (2–3 times/week or daily) • Classroom intervention (1:1, 1:4, 1:10) with teacher and parents • Counseling and training of parents via discussions, family meetings, home visits, referrals to doctors and other paramedical professionals and use of problem solving approaches • Medical intervention for symptoms of epilepsy or dystonia • Ignoring counterproductive attention-getting behaviours and rewarding cooperation with simple praise • Physical and verbal prompting	In classroom 2–3 hrs/day, 5 days a week. At home: Daily	Verbal classroom sessions with parents and children	Weekly assessment
**Lakhan (2014)**	N/A	Children with ID and their parents Dropout Rate: 65%	Behavioural Assessment Scale for Indian Children with Mental Retardation (BASIC‑MR); Developmental Screening Test (DST); VSMS	**For Parents** Community-based rehabilitation program staff **For Children:** Professionals specializing in ID	• Restructuring the environment • Extinction • Token economy • Over correction • Response cost • Differential reinforcement for incompatible/alternate behaviour • Differential reinforcement for low-frequency behaviour • Differential reinforcement for other • Physical restraining • Study participants given daily schedule to follow and involved in household activities wherever possible	1 year	Training course at NGO Ashagram Trust (AGT)	Every month by CBR workers Every 3 months by professionals
**Russell et al. (2004)**	Individualized training plan based on the child’s adaptive behaviour delays adapted from the Progress Assessment Charts (UK) or the Carolina Curriculum for Infant and Toddlers with Special Needs (USA)	Children with ID and their parents Dropout Rate: 9%	Binet–Kamath Scale of Intelligence; VSMS; Parental Attitude Scale towards Management of Intellectual Disability	**For Parents:** Special Educators, Psychologists, Occupational Therapists, Speech Therapist and Psychiatrist **For Children:** • Psychologist to assess adaptive behaviour • Adaptive behaviour training by team of Special Educators, Psychologists, Occupational Therapists, Speech Therapist and Psychiatrist	• Interactive group psychoeducation • Multimodal adaptive behaviour training plus didactic lectures • Training in self-care skills, home and independent living skills, social skills, sensory motor skills, language, concepts and control of problem behaviours using special education and behavioural techniques	5 session per week for 12 weeks	• Interactive group psychoeducation closed group sessions • Didactic lectures	Weekly
**Russell et al. (1999)**	N/A	Children with ID and their parents Screened and 57 ID children and their parents recruited; Dropout Rate: 9%	Parental Attitude Scale towards the Management of Intellectual Disability	**For Parents:** Psychxque ologists, Special Educators **For Children:** Occupational Therapist (clinical exam),Psychiatrist (interview/discussion)	• Interactive Group psychoeducation (IGP) in closed group sessions twice/week for 10 weeks • One-hour sessions on aspects of child-rearing skills, developmental milestones and delays, common causes of intellectual disability, comorbidities, functional skills, problem behaviours, behavioural techniques, sexuality and marriage and legal and social support systems • Child trainings in self-care, social and pre-vocational skills, and control of problem behaviour using special education and behavioural techniques	Entire training duration: 12 weeks. (Experimental and control therapy for parents: 10 weeks. Other trainings: 2 weeks)	• Closed group interactive group psychoeducation (IGP). • Didactic lecture	At 12 week follow-up
**Mohsin et al. (2011)**	N/A	Children with ID and their parents Dropout Rate: N/A		Trainer	Three phases: • Phase-1: parent observation of frequently performed daily activities, special event (e.g. birthday) • Phase-2: Parents asked to describe environment, sub-environment and related tasks, which the child is unable to perform and draw a list of desired tasks to be learnt by their child • Phase-3: Develop education program for children	15 days	Face-to-face interview with parents regarding child performance	At 15 day follow-up Endline survey conducted since baseline to 15 days follow-up
**Narayanan et al. (1988)**	N/A	Children with ID (IQ<50) and their families Dropout Rate: N/A	Mental retardation training module developed by NIMHANS	Psychiatrist, Clinical Psychologist, Psychiatric Social Worker, Speech Therapist, Psychiatric Nurse	• Intensive counseling for parents • Parents trained in elements of behaviour modification • Child-specific therapies depending on needs (i.e., speech therapy, occupational therapy/physiotherapy, behaviour therapy) • 35 children (33%) received medication for epilepsy or psychiatric/medical indications • 64 (60.4%) parents received instruction in sensory motor stimulation, and 72 (67.9%) training to support self-help skills • Genetic counselling offered to 5 (4.7%) parents	2 weeks	Counselling, training of the parents (instruction, discussion, demonstration, and in-vivo feed-back) in techniques of multisensory stimulation, speech, motor, and self-help skills training, behaviour modification and medical management	After 2 week follow-up

#### (iii) Methodologies

Two of the studies were RCTs and four were pre-post studies. One pre-post study of a community-based intervention programme for parents of children or adolescents with ID in India had a large sample size and included follow up after the intervention program, making it generalizable to a wider range of populations [83]. In another pre-post study, the duration of the in-patient stay was much shorter, and in the other two pre-post studies the sample sizes were very small, [51, 84, 85] (**Table 2**).

#### (iv) Quality

According to Kmet criteria, three studies were of strong quality, two were good, and one was of adequate quality. The two RCTs described the eligible populations, tools and intervention procedures in details [49, 81]. The sample size of three pre-post studies was quite low and it is difficult to compare the effectiveness of these studies [51, 84, 85] (**S1 Table**).

#### (v) Adaptation process of the intervention programmes

Only two studies mentioned adaptation of an intervention from a foreign country. In Paul, Jacob, et al. (1999), the training plan for children was adapted from both Progress Assessment Chart (UK) and the Carolina Curriculum for Infant and Toddlers with Special Needs (Baltimore, USA) [81]. In addition, Kurani et al. (2009) [51] adapted the Portage Parent Program, widely used in India since 1980, to conduct an effective program for native language speakers. This study adopted the Parental Involvement/Engagement Scale for interpreting the parental involvement in terms of child-oriented teaching parents, planning and cooperation by both teachers and parents, child involvement in naturally occurring social settings, within school, home and community [51]. Lakhan (2014) adopted the Behavioural Assessment Scales for Indian children with Mental Retardation (BASIC-MR), which lists 75 behavioural problems in 10 domains [83]. The BASIC-MR was developed locally in India for children in special needs schools [86] (**Table 2**).

#### (vi) Components of interventions

**Parent components:** Five of the studies reported on parent-focused interventions. Mohsin et al. (2011) implemented a functional skills intervention program that included a diaries method to identify functional tasks and a Weekly Evolution Report (WER) to deliver the teaching or training at the next home visit [85]. In addition to home visits and family meetings, Kurani et al. (2009) implemented therapeutic and classroom interventions, along with parent counseling and referrals to doctors [51]. Narayanan et al. (1988) implemented an intervention where parents were trained on sensory-motor stimulation and development of motor, language, and self-help skills [84]. Lakhan (2014) trained parents to manage hyperactive behaviours, through praising a child’s good activities, over-correction of children’s misbehavior, training on physical restraining if required, and other techniques [83]. Parents were also encouraged to involve their children in household activities wherever possible [71]. Interactive Group Psycho education (IGP) was conducted by psychologists in Paul, Jacob et al. (1999) to teach parents child-rearing skills, developmental milestones and delays, common causes of intellectual disability, comorbidities, functional skills, problem behaviour, and behavioural intervention techniques, as well as to provide information on sexuality and marriage and legal and social support systems. The one study conducted in Pakistan included parent education about ASD and formal training in a developmental approach to address child symptoms during home visits [81] (**Table 2**) and **(S2.2 Table in S2 Table**).

**Child and components:** Five studies focused interventions on the child. Two studies implemented medication interventions in addition to therapeutic measures [33, 73]. Two studies trained children or adolescents in self-care, social skills, pre-vocational skills, and control of problematic behaviours [48, 74] **(Table 2**).

#### (vii) Modality of the intervention

**Setting:** Three studies were conducted in clinic/hospital settings, two in community settings and only one in a school setting. **Duration:** The duration of the intervention ranged from one week to one year. **Service providers:** Three studies reported that teachers or special educators and occupational therapists provided the intervention followed by psychiatrists (3), psychologists (2), workers from community-based rehabilitation programmes (2) and field assistants (1). In addition, nurses (1), specialists (e.g., speech-language therapists and professionals in ID also contributed. **Ways of delivering:** The intervention programmes were mainly delivered in closed group sessions (4), although a camp setting (1) and one-on-one sessions (1) were also applied. **Time to evaluation or end-line:** The time from intervention end to evaluation ranged from a minimum of one week to a maximum three months. **Dropout rate**: Three studies registered the dropout rate of participants, and this ranged from 9% to 65% [49, 81, 83] (**Table 2**).

#### (viii) Changes in the outcome

**Parent outcome:** All 6 studies reported a parent outcome, including significant improvements in parent attitude and knowledge. In addition, one study highlighted improved parent motivation to teach their children [73] (**Table 2**). Out of five studies on ID, only one study assessed impact on parental knowledge using a total parental attitude scale.

**Child and adolescent outcome:** Three studies reported on child outcomes. One intervention based on IGP reduced the degree of disability and improved child self-help, eating and socialization skills [49, 81]. Two pre-post studies reported improvements in motor, cognitive, social and language development, as well as reduction of violent or self-injurious behaviour and temper tantrums [51, 83] (**Table 2**).

### ADHD (Attention Deficit Hyperactivity Disorder)

#### (ⅰ) Study characteristics

Four studies were conducted among parents of children or adolescents with ADHD, two in Pakistan and two in India.

#### (ⅱ) Tools used

For participant inclusion, two studies used the Disruptive Behavior Disorders Rating scale (Barkley, 1997) to gather symptoms and did not further discuss diagnostic criteria, one used DSM-V diagnostic criteria implemented by a consulting psychiatrist, and one reported using ICD-10 criteria. Assessment tools utilized included the Disruptive Behaviour Disorders Rating Scale (DBDRS) for ADHD screening and the Home Situations Questionnaire (HSQ) and School Situations Questionnaire (SSQ) for assessing the severity of ADHD symptoms [16, 87, 88], as well as the Color Cancellation test was used to assess attention, concentration and impulsivity, the ADHD rating scale to measure the severity of ADHD and an academic performance rating scale to asses child academic performance, Barkley’s School Situations Questionnaire (BSSQ) to assess situational variations in child behaviour, and the Family assessment Schedule to assess family stress [89] (**Table 3**).

**Table 3:** Characteristics of the included studies for children and adolescents with Attention Deficit Hyperactivity Disorder (ADHD).

Author, year of publication	The process of adaptation	Description of participants	Tools for assessment	Service providers	Components of the intervention	Duration of each treatment/intervention	Modality of training process	Times of evaluation
**Malik and Tariq (2014)**	• Parent training manual, adapted from *Guillemin*, *Bombardier*, *& Beaton*, *1993* • Feedback from program developer Barkley (1997) and other experts, as well as extensive literature review and examination of other studies with published cultural adaptation procedures aided with adaptation planning.	Children with ADHD and their parents Dropout Rate: 39%	DBDRS; School Situation Questionnaire (SSQ); Home Situations Questionnaire (HSQ)	Therapist	• Parents trained in more effective ways to attend to child’s behaviour and enhance the value of their attention • Attend to child compliance and independent play • Parents set up a home token economy to provide external reinforcers for activities not intrinsically motivating, such as home chores. • Implement time out for non-compliance • Extend time out for additional non-compliant behaviours • Parents taught to extrapolate home management methods to troublesome public places, such as stores, church and restaurants • Improve child school behaviour from home	4 months	• Training conducted in group sessions • Visual package provided to parents to watch at home at beginning of training • Portions of package utilized during group sessions with real-life examples of challenges families often encounter	After 4 months of intervention
**Malik et al. (2017)**	• All rating scales translated into Urdu using the guidelines provided by Guillemin, Bombardier, and Beaton (1993) • Feedback from the program developer Barkley (1997) and other experts, as well as extensive literature review and examination of other studies with published cultural adaptation procedures aided with adaptation planning.	Children with ADHD and their parents	Disruptive Behaviour Disorders Rating Scales (DBDRS); SSQ; HSQ; Developmental History Form	Therapist, Psychology Interns and Graduate Students	• Same as Malik and Tariq (2014)	10 weeks	• Same as Malik and Tariq (2014)	After 10 weeks of intervention
**Shah et al. (2019)**	N/A	Parents of children with ADHD, Grandparents attended some sessions Dropout Rate: 56.4%	5-point Likert scale;10-point visual analogue scale; Vanderbilt ADHD Diagnostic Parent Rating Scale (VADPRS)	Clinicians	Specific skill-building to handle consequences of ADHD effectively and confidently	10 weeks	• Group parent training sessions (10) of 90 minutes duration each • Delivered by video through Zoom • Weekly reminders regarding homework and upcoming sessions • Face-to-Face intervention done traditionally • Hospital visits once in 4–8 weeks	After 8 weeks of intervention
**Rejani et al. (2012)**	• Parent training conducted using the manual: Defiant Children-A clinician’s manual for parent training (Barkley, 1997)	Parents and children with ADHD Dropout Rate: 43%	ADHD Rating Scale; Academic Performance Rating Scale; SSQ; HSQ; Family Assessment Schedule; Colour Cancellation Test	Psychiatry consultant, Therapist	• Behavioural management, and tasks for enhancing parental attending skills • Establish home token systems, using response cost • Improve school behaviour, • Manage child’s behaviour in public places • Plan for future behaviour problems • Attention enhancement training: Package included coloring, grain sorting, clay modeling, mazes, beading and matching figures. Based on empirical evidence for improving attention deficit and impulsivity. • Medication management: methylphenidate or clonidine, choice by Psychiatry Consultant. Consultation frequency similar for both groups	10 weeks	• Tasks introduced in weekly sessions • Group discussion spread over ten weekly sessions of one-hour duration each	After 10 weeks of intervention.

#### (iii) Methodologies

One study implemented a quasi-experimental design; the rest were pre-post studies. The quasi-experimental study was completed in Pakistan and included only participants from the city of Islamabad [16]. Among the pre-post studies, a multimodal intervention programme was conducted in India in which Group I received medical management with parent counseling and Group II, the multimodal group, received routine medical management, parent training, and attention enhancement training [87]. Two studies evaluated a behavioural parent training programme and trained parents by multi-point video-conferencing [90] (**Table 3**).

#### (iv) Quality

One study was of strong quality, and three studies were of adequate quality using the Kmet criteria. Methodological limitations of two of the three pre-post studies included a small sample size, outcomes measured only by parent and teacher report [87, 89] and a high dropout rate in one study [90]. One study did not report on blinding of investigators, so the outcome could be exposed to bias [90]. In the quasi-experimental study, participants were not randomly assigned to treatment and control groups, and included participants from the city, hence it is less likely to be generalizable to rural communities [16]. Finally, one pre-post study was more promising due to the involvement of random allocation and parallel design [82] (**Table 3**).

#### (v) Adaptation process of the intervention programmes

Two studies adapted a manual from Barkley Behavioural Parental Program (1997) [88]. The adaptation process involved translation and extensive literature reviews to support cultural adaptation and validation [16, 87] (**Table 3**).

#### (vi) Components of the intervention

**Parent components:** All 4 programs reported on parent intervention. Two intervention programmes used a parent training manual adapted from Defiant Children: A Clinician’s Manual for Assessment and Parent Training, Barkley Behaviour Parental Program (1997) [88]. The manual contains information about ADHD, such as the causes of defiant behaviour in children with ADHD and what factors affect it [16, 91]. One intervention study trained parents to manage child behaviour problems using a visual educational package [16]. Another intervention programme used the same training manual, but a ten-step programme was set up to teach principles of behavioural management and tasks for parental attending skills [87]. This programme also included Attention Enhancement Training and Medication Management. Finally, Shah et al. (2019) incorporated culturally relevant methods and content to improve awareness, parent-child relations, family environment, and specific skills to handle consequences of ADHD effectively and confidently [90] (**Table 3**).

**Child and adolescent components:** Only one of the four studies conducted a programme designed to improve the child’s attention, communication, social skills, and behaviour. Rejani et al. (2012) used the manual of Defiant Children (Barkley, 1997) to create situations during family daily activities to improve attention deficit and impulsivity. These activities used material available at home, such as grain sorting, clay modelling, mazes, beading and matching figures [87, 88]. Medication management under the supervision of the psychiatry consultant was an additional component of this study (**Table 3**).

#### (vii) Modality of the interventions

**Settings:** All four studies were conducted in clinic/hospital settings [16, 87, 89, 90]. **Duration:** The length of intervention varied from 2.5 to four months [16, 87, 89, 90]. **Service providers:** Interventions were provided by therapists (2), clinicians (1), psychiatry consultant (1), psychology interns and graduate students (1). **Ways of delivering:** The intervention programmes were delivered by closed group sessions, one-on-one sessions and home-based sessions [16, 87, 89, 90] and through electronic media-like video conferencing [90]. **Time to evaluation or end-line:** Evaluations were conducted after ten weeks to four months of intervention. **Dropout rate:** Three out of four studies mentioned a dropout rate, which ranged from 43% to 56.4% (**Table 3** and **S2.3 Table in S2 Table**).

#### (viii) Changes in outcome

**Parent outcome:** All four studies reported on parent outcomes. All pre-post intervention studies showed improvement in parent behaviours after the training. Two studies reported significant improvement in parental behaviour, such as child rearing techniques, knowledge towards ADHD, attitude towards management of ADHD [89, 90]. Other studies reported caregivers were more aware about the symptoms, causes, consequences and course of ADHD, had reduced guilt associated with causation of ADHD, were less likely to blame the child and showed improved parent-child relationships [16, 90] (**Table 3**). Out of four studies on ADHD no studies assessed parental knowledge.

**Child and adolescent outcome:** All four studies reported child outcomes. The quasi-experimental study reduced in attention, oppositional defiant disorder (ODD), and conduct disorder (CD) symptoms [89]. Another study showed significantly reduced child ADHD-related impairment [16]. The same study and another also reported significant improvements in behavioural problems at school, learning ability, impulse control, and academic performance, as well as reduced social withdrawal [16, 89]. The final study reported reduced interpersonal problems at home, and improved communication style, self-competency and ability to convey information clearly to teachers and tutors [90] (**Table 3**).

### Cerebral palsy

#### (i) Study characteristics

Four studies were conducted between 2000 and 2015 among parents of children with CP in India (n = 3) and Bangladesh (n = 1).

#### (ii) Tools used

For participant inclusion, all children or adolescent were diagnosed clinically. One study utilized the WHO International Classification of Impairments, Disabilities and Handicap. The remaining three used unspecified clinical diagnostic criteria. Assessment tools utilized focused on child behaviour, maternal adaptation, parental knowledge and family support (**Table 4**).

**Table 4:** Characteristics of the included studies for children and adolescents with Cerebral Palsy (CP).

Author, year of publication	The process of adaptation	Description of participants	Tools for assessment	Service providers	Components of the intervention	Duration of each treatment/ intervention	Modality of training process	Times of evaluation
**Maiya et al. (2015)**	N/A	Children with CP and their parents Dropout Rate: N/A	Examination of child oral and gingival hygiene	Trainer	• Taught importance of oral hygiene maintenance and knowledge regarding different preventive home care measures Taught importance of oral hygiene maintenance and knowledge regarding different preventive home care measures provided • Taught horizontal tooth brushing with manual toothbrush and the correct usage of powered toothbrush with pea-sized amount of fluoridated toothpaste advised to be performed twice daily by the parents/caregivers. • Provided manual toothbrush with fluoridated toothpaste; fluoridated toothpaste and CHX spray; Powered toothbrush with fluoridated toothpaste; Powered toothbrush with fluoridated toothpaste and CHX spray	6 weeks	Health education program to parents/caregivers/institution staff	At end of intervention
**Arora et al. (2014)**	N/A	Children with CP and their parents Dropout Rate: N/A	• Parental knowledge regarding CP: • name of the disease • probable aetiology • treatment options • rehabilitation options/potential • Training for child to perform activities of daily living • Parental involvement in training • Parent’s knowledge regarding child development	Pediatrician	• Parents shown an educational film in Hindi language about: • CP aetiology and management • Role of parents in managing CP • Eight spheres of development and how they are involved in CP	1 day	One educational film on CP	One week after intervention
**Karande et al. (2008)**	N/A	Children with CP and their parents Dropout Rate: 18.7%	Questionnaire about basic issues for children with CP	Pediatrician	• Disclosure of diagnosis of CP to the parent(s) • Education about the “core basic issues” of CP • cause • child’s patterns of movement and muscle tone • it is treatable • frequency and duration of early intervention therapy • Referral for early intervention therapy • Importance of following up regularly with a pediatrician • Prevention of cerebral palsy in next child	1 day	• Educational program through practical session using “flash cards” at the clinic • 25 minute duration	After 3 months
**McConachie et al. (2000)**	N/A	Children with CP and their parents Dropout Rate: 36.9%	The Independent Behaviour Assessment Scale(IBAS); Self-Report Questionnaire; Maternal Adaptation to the Child (Judson Scale); Family Support Scale; Parental Knowledge Questionnaire	Therapist	• Health advice to parents to improve child motor, speech and language, and cognitive skills • Practice of daily living skills • Developmental activities • Regular attendance intervention • Child health assessment	12 months	**Distance Training Packages:** • One to two hour face-to-face session • Some pictorial manuals provided to take home **Mother-Child Group:** • Center-based, face-to-face sessions daily • Led by a therapist with physiotherapy training • Involved both children and parents **Health Advice Group**: • Nutritional advice • Vitamin supplements	9 to 12 onths after initial assessment

CP: Cerebral Palsy; CHX: Chlorhexidine; IBAS: Independent Behaviour Assessment Scale.

#### (iii) Methodologies

This review included one RCT and three pre-post intervention studies. Of the pre-post studies, one was conducted in two special schools in the Bangalore city, India, and children were randomly divided into four groups, although the sample size per group was small (16 children per group) [66]. Another pre-post study was conducted in the developmental clinic with pediatrician completing detailed history and neurological examination to confirm CP. The third pre-post study was conducted in a tertiary care hospital with children recently diagnosed (≤1 month) with CP [92]. The only RCT was conducted in Bangladesh and included two arms for children living in urban areas and two arms for children in rural areas. The number of participants was small, with losses to follow up in both arms [48] (**Table 4**).

#### (iv) Quality

The Kmet quality ratings of the CP-related studies were the lowest of the four reviewed NDDs. Only one study was of good quality, and three were reported to be of adequate quality. The RCT clearly described the process of recruiting study participants, but did not discuss participants who dropped out or declined to take part in the study. Despite small sample size, it demonstrated positive outcomes of parent-mediated interventions [48] (**Table 4**).

#### (v) Adaptation process of the intervention programmes

None of the interventions were adapted from programmes in other countries.

#### (vi) Components of the interventions

**Parent components:** In a randomized open-blinded study by Maiya et al. (2015), parents were taught different preventive home care measures through educational programmes [66]. Two other studies, Karande et al. (2008) and Arora et al. (2014), targeted parental knowledge about CP, its probable a etiology, and available treatment and rehabilitation options [92, 93]. McConachie, et al. (2000) provided outreach training to improve parental knowledge [48] (**Table 4** and **S2.4 Table in S2 Table**).

**Child and adolescent components:** One study include educational material about oral and gingival hygiene (60). The majority of the interventions included components to improve child motor, speech-language and cognitive skills, as well as parent-child interactions (**Table 4** and **S2.4 Table in S2 Table**).

#### (vii) Modality of the interventions

**Settings:** Studies were conducted in hospital, school and community settings. **Duration**: The length of intervention varied from one day to two months. **Service providers:** Interventions were provided by a trainer (1), a pediatrician (1), or a therapist (1) through film, educational programmes, or mother-child groups. **Ways of delivering:** The interventions were provided by closed group sessions, one-to-one session and practical sessions through educational film, flash card, pictorial manuals, or health advice to the parents [48, 66]. **Time to evaluation or end-line:** The time from intervention to evaluation varied from one week to 12 months. **Dropout rate:** Half the studies stated the dropout rate, which ranged from 19% to 36% (**Table 4**).

#### (viii) Changes in outcomes

**Parent outcome:** All articles reviewed reported parental outcomes. Three studies increased parent knowledge [34, 92]. One study reported improvements in knowledge regarding maintaining oral hygiene [66] (**Table 4**). Out of four studies on CP, three recorded parental knowledge and used locally developed questionnaires specific to the studies.

**Child and adolescent outcome:** One RCT showed improvement in participant oral hygiene and gingival health status, an important part of the activity of daily living [48] (**Table 4**).

## Discussion

The current systematic review reviewed the published literature on parent-mediated intervention programmes designed to change outcomes for parents or children or adolescents with four common NDDs (ASD, ID, ADHD, and CP) in South Asian countries. It appraised 23 relevant studies to assess the acceptability of implementation. One third (8) of the intervention programmes (mostly for ASD, ADHD and ID) adapted and culturally validated successful intervention programme from high-income countries, mostly from the United Kingdom and Unites States of America. The majority of the intervention programmes were implemented in a clinic or hospital setting and were provided for an average of 12 to 24 weeks. Parents were primarily trained by special educators, most commonly a speech or occupational therapist.

In South Asian countries, where availability of psychiatrists or psychologists is inadequate [94], such special educators are a particular resource for parent training to fill the large gaps in service availability for children or adolescents with NDDs. Most parent trainings were provided in groups, and different visual aids were used. One potential benefit of parent groups is that they can build a sense of inclusion and provide additional learning through parent-to-parent exchanges and have the potential to reduce parenting stress [95]. Parents of children or adolescents with NDD are subject to social stigma, which may limit their accessing of needed care. Peer support from other parents can create a supportive environment and raise social awareness, particularly in places where services are limited, such as in South Asia. In terms of parental outcome, the interventions showed improvements primarily in knowledge, attitude and management of the child or adolescents at home across all the disorders. Improvements in parent-child interaction were also highlighted in many of the studies. Among the children or adolescents, improvements were seen in activities of daily living (e.g., maintaining oral hygiene and self-care), communication and play skills. Reductions in repetitive and disruptive behaviours were also reported.

Findings from the ASD literature suggest that parent-mediated interventions can improve parental knowledge and influence social behaviour and communication skills of children and adolescents. Cross-study comparisons were not possible due to the wide variation in the intervention components, duration, delivery modalities and outcomes measured. Additionally, parent-mediated interventions had a significant impact on developmental domains, including cognition, socio-emotional, and activity of daily living, as well as improvement of sensory processing abnormalities in children or adolescents with ASD [33, 77]. Similar findings have also been shown in studies from high income countries, including improvement of parent-child interaction, parental behaviour with children, and child social interaction skills [96–98]. Positive findings were not consistently observed across all the studies and further investigation will be required to more fully assess the effectiveness of these interventions. Approximately three-fourths of parents completed the intervention programmes, suggesting a potential demand for parent-mediated interventions, with one of 5 studies reporting completions rates having very high drop-out rate (65%). Over 90% of parents in the other studies completed the full intervention.

Likewise in studies for children or adolescents with ID, parent-child communication and parent motivation to teach their children both increased. In addition, specific behavioral improvements were reported among children in rural areas, where centralized or specialized programmes are even less available, as well as improvement in motor and social skills [99, 100]. These findings are consistent with those of studies from the other countries [101, 102]. More than two-thirds of parents of children or adolescents with ID completed the intervention programmes, although the one study of longest duration (1 year) had only one-third of families complete the study.

For children or adolescents with ADHD, parent-mediated interventions showed positive impacts by reducing child inattention, hyperactivity, oppositional defiant behaviours and conduct disorder symptoms [16, 87]. In fact, parent involvement has been shown to be essential for all behavioural treatments of ADHD, although the type of parental involvements may differ [16]. Family completion rates for children with ADHD were the lowest of all, with just over half of families completing the intervention period on average. This brings into question the feasibility of these programs in South Asia as currently administered and suggests more work will be needed in this area.

Finally for children or adolescents with CP, parent-mediated interventions improved parental knowledge about CP and its causes and management. Improvements in child adaptive skills were also seen, consistent with findings from developed countries [103]. More than two-thirds of enrolled families completed these interventions. However, only one of the reviewed studies as of good quality and none were strong, so findings of these studies need to be interpreted with caution [66]. Importantly, the reviewed literature clearly demonstrated that parents of children with CP in South Asia still have very little information about CP, associated conditions and management, a fact that hinders their ability to support their children appropriately [104].

The burden of NDDs is higher in South Asian countries, and high-quality evidence for appropriate parent-mediated interventions is still lacking. Only seven out of twenty-three studies in this review were of strong quality, and most of these were done in the areas of ASD and ID. Several pre-post studies had methodological weakness including subjective assessment of the parent’s motivations, inconsistent use of random treatment allocation, small sample sizes, and outcomes measured by non-standardized approaches. In addition, the vast majority of studies were conducted in India (eighteen), with only four from Pakistan and one from Bangladesh, thus limiting the generalizability of findings across the region. The number of community-based RCT studies was also very limited, making generalization of findings even more difficult. Nevertheless, even with the high variability in study setting, intervention and the assessment tools used, many interventions were validated and customized in the local context, suggesting that these may be implementable in other South Asian countries. There are likely be several reasons for the limited number of studies within South Asian countries, including inadequate knowledge about NDD’s, lack of support centres and absence of appropriate government supports for programmatic implementation [105].

The current systematic review identified several opportunities for further research. Futures studies would benefit from more clearly outlining diagnostic assessment criteria for study inclusion, since misclassification of an NDD (e.g., by use of a tool not validated or appropriate adapted to the local setting) could alter the study outcome. Although some prevalence-based surveys are available, implementation research for parent-mediated intervention programmes is notably limited in this region [10, 14, 15]. Programs that have shown success on a small scale or under controlled conditions could be expanded to larger samples as a step toward scaling. Systematic monitoring and evaluation of these programmes will be extremely important [106]. In high-income countries, it is a common practice to involve parents and family members in interventions for children or adolescents with NDDs, equipping them with much needed skills. Several parenting programmes have shown effectiveness in high-income countries, but the feasibility and acceptability of these intervention programmes in low-resource country settings should be further investigated. In the context of South Asian countries, large scale intervention programmes that also involve feasibility assessments are crucial for the management of NDDs. Furthermore, cost-effectiveness analyses were missing from the reviewed literature and will be important to guide decision makers on intervention choice and effective use of limited resources. Additionally, NDDs significantly impact the quality of life of children and families [107]. However, none of the reviewed studies reported on quality of life outcomes for children and parents following the parent-mediated interventions constituting a significant and important knowledge gap. Only six studies assessed impact of parental training on parental knowledge. This is a missed opportunity for the studies in which the researchers did not attempt to assess impact on parental knowledge following training. Finally, parent-to-parent programmes have shown success in other settings [23, 108], and should also be explored to disseminate evidence-based interventions in South Asian countries in a more broader approach. This review is a valuable first step toward understanding the acceptability of implementing these programmes in South Asia and provides guidance for future large scale studies to assess programme effectiveness.

### Limitations

Although the current review was extensive and systematic, it has several limitations. All the studies retrieved from the search were published in English, although the search did not restrict on the basis of language. Thus there might be publication bias in the field that could impact the generalizability of findings. This review did not consider the grey literature, which may also introduce bias. The assessment tools, study settings, outcomes measured, and delivery mode of intervention in the reviewed studies were heterogeneous, which complicated our ability to interpret and generalize the findings of parent-mediated intervention programs. The heterogeneity of the twenty-three studies included in the review limited the ability to make comprehensive recommendations and prevented meta-analysis; however, findings do highlight the potential of benefit and provide a guide for next steps in research on parent-mediated interventions for children with NDDs. Despite these limitations, our review suggests that parent-mediated programmes implemented in South Asia have a positive impact on parental knowledge, attitude, and performance, as well as on social communication, behaviour and parent-child interactions of children or adolescents.

This systematic review has several strengths. This is the first systematic review focused on parent-mediated intervention programmes targeting children with NDDs in the South Asian context. Its methodology was rigorous and followed the Preferred Reporting Items for Systematic Reviews and Meta-Analyses (PRISMA) approach. The aggregated evidence from this review generated important research questions for future study and will help guide investigators, funding agencies, and government policy makers.

## Conclusions

Overall, evidence from this review suggests that parent-mediated intervention programmes have a promising role in the management of children with ASD, ID, ADHD, and CP in South Asian countries, particularly when programmes are customized to the need of the local context. Findings can inform development of new interventions, as well as further study of available ones. Nevertheless, further work and large-scale studies are necessary to identify factors that contribute to the success of these intervention programmes.

## Supporting information

S1 Checklist PRISMA 2009 checklist. (DOCX)

S1 Table Quality of the studies according to the Kmet criteria. (DOCX)

S2 Table Description of the intervention programs. (DOCX)

S3 Table Search terms for each database. (DOC)
