# Supplementary material for: Parent mediated intervention programmes for children and adolescents with neurodevelopmental disorders in South Asia: A systematic review
Source: PLoS One. 2021 Mar 11;16(3):e0247432. doi: 10.1371/journal.pone.0247432 (PMC7951928; doi:10.1371/journal.pone.0247432)
Supplement: S1 Table — (DOCX) [file pone.0247432.s002.docx]

**S1 Table.** Quality of the studies according to the Kmet criteria

| Study | Type of NDD | Study design | Population Eligible and Defined | Allocation to Intervention (or Comparison) | | Outcomes | Analysis |
| --- | --- | --- | --- | --- | --- | --- | --- |
|  |  |  |  | Allocation | Retention |  |  |
| Brezis, Weisner et al. (2015) | ASD | Pre-post | + | ++ | _ | ++ | ++ |
| Rahman, Divan et al. (2016) | ASD | RCT | ++ | ++ | ++ | ++ | ++ |
| Nair, Russell et al. (2014) | ASD | Pre-post | + | NR | NR | ++ | + |
| Juneja, Mukherjee et al. (2012) | ASD | Pre-post | ++ | NR | NR | ++ | + |
| Krishnan, Nesh et al. (2016) | ASD | Pre-post | + | NR | NR | ++ | ++ |
| Louis and Kumar (2015) | ASD | RCT | ++ | NR | NR | ++ | + |
| Divan, Vajaratkar et al. (2019) | ASD | RCT | ++ | + | + | ++ | ++ |
| Manohar, Kandasamy et al. (2019) | ASD | RCT | ++ | + | _ | ++ | ++ |
| Padmanabha, Singhi et al. (2019) | ASD | RCT | ++ | ++ | + | ++ | ++ |
| Lakhan (2014) | ID | Pre-post | + | NR | NR | ++ | ++ |
| Kurani, Nerurka et al. (2009) | ID | Pre-post | + | NR | NR | ++ | ++ |
| Russell, al John et al. (1999) | ID | RCT | ++ | ++ | ++ | ++ | ++ |
| Mohsin, Kahn et al. (2011) | ID | Pre-post | _ | NR | ++ | ++ | + |
| Russell, John et al. (2004) | ID | RCT | ++ | ++ | ++ | ++ | ++ |
| Narayanan, Girimaji et al. (1988) | ID | Pre-post | + | NR | + | + | + |
| Malik, Rooney et al. (2017) | ADHD | Quasi-experimental | + | ++ | ++ | ++ | ++ |
| Malik and Tariq (2014) | ADHD | Pre-post | ++ | _ | NR | ++ | ++ |
| Rejani, Oommen et al. (2012) | ADHD | Pre-post | ++ | ++ | ++ | ++ | ++ |
| Shah, Chakrabarti et al. (2019). | ADHD | Pre-Post | ++ | - | - | + | + |
| Maiya, Shetty et al. (2015) | Cerebral Palsy | Pre-post | ++ | _ | ++ | + | ++ |
| Arora, Aggarwal et al. (2014) | Cerebral Palsy | Pre-post | + | _ | NR | + | + |
| Karande, Patil et al. (2008) | Cerebral Palsy | Pre-post | _ | _ | NR | + | ++ |
| McConachie, Huq et al. (2000) | Cerebral Palsy | RCT | + | ++ | ++ | ++ | ++ |
