# Supplementary material for: Parent mediated intervention programmes for children and adolescents with neurodevelopmental disorders in South Asia: A systematic review
Source: PLoS One. 2021 Mar 11;16(3):e0247432. doi: 10.1371/journal.pone.0247432 (PMC7951928; doi:10.1371/journal.pone.0247432)
Supplement: S2 Table — (DOCX) [file pone.0247432.s003.docx]

**S2 Table:** Description of the intervention programs

**S2.1 Table:** Description of intervention programs for the children with ASD

| **Study(year)(Type of NDD) and Country** | **Structure of Intervention program** |
| --- | --- |
| **Krishnan, Nesh et al. (2016)**  (ASD)  India | **Structure:**   - Clinic-based, multi-component early intervention package with2components:  1. The standard intervention protocolthat   included training in self-care skills, social skills and control of problem behaviour using  special education and behavioral techniques   1. Parents were taught about ASD, using an interactive group psycho-educational technique. The Psycho-Educational Profile-Revised (PEP-R) intervention included teaching activities and treatment for children with ASDand developmentally disability, teaching strategies for parents and for individual assessment 2. The Carolina Curriculum for Infant and Toddlers with Special Needs (CCITSN) module used a developmental approach to address ASD with focus on cognitive and motor skills, communication and social adaptation.   **Method of Delivery:**   - Each session started with a 10-minutebriefing about goals for the day. - The children were then engaged in play routines and social stories - Each parent–child dyad received applied behavior analysis aimed at improving behavioral control and interactive skills of parents using principles of rewarding and guided practice. - Intervention was in the form of closed group sessions conducted five times a week for 4 hours. |
| **Rahman, Divan et al. (2016)**  (ASD)  India and Pakistan | **Structure:**   - A naturalistic approach for scaffolding and developing communication skills - Used parent-mediated Intervention for Autism Spectrum Disorder in south Asia (PASS) - In the experimental group, PASS was delivered individually.   **Method of Delivery:**   - One-to-one clinic or home sessions between health worker and parent with the child present - Health workers were supported with semi-structured scripts. - One hour sessions every 2 weeks for 6 months (12 sessions) - Initial home visit from non-specialist health worker, supported by a local specialist, explored parents’ beliefs about the nature and origin of ASD and other factors that might affect engagement, including individual learning styles of the target parent. - Each parent and child session was videotaped and reviewed in detail with parents for progress since last session, fidelity to treatment goals, and planning of the next steps. - Parents were asked to spend 30 minutes a day between clinic sessions practicing predefined strategies at home and encouraged to keep a daily record of their achievements. |
| **Brezis, Weisner et al. (2015)**  (ASD)  India | **Structure:**   - Incorporated Western ASD-training methods (e.g., TEACCH, Applied Behavioral Analysis, Floor time) with cultural adaptations - A narrative interview method “The Five Minute Speech Sample (FMSS)” was used as the assessment. Parents were prompted to speak about their children and their relationships with them for five uninterrupted minutes.   **Method of Delivery:**   - Training included daily group and one-on-one activities with the children (led by the parents) and group discussions for the parents. |
| **Louis and Kumar (2015)**  (ASD)  India | **Structure:**   - Fathers were taught to use a list of words during play and to reinforce responses of attachment and reciprocity. - Fathers were taught simple messages to help the child perform activities of daily living.   **Method of Delivery:**   - Fathers in the treatment group attended a clinic based programconsisting of1-hour sessions three times during the intervention period. - Fathers observed how the therapist interacted with the child, engaged the child on a one-to-one basis, and then demonstrated how they would engage the child at home. - Father’s participation was mandatory. |
| **Nair, Russell et al. (2014)**  (ASD)  India | **Structure:**   - Parents trained to identify developmental age of child, using an intervention kit assembled of inexpensive materials available at home or the local market. - Parents educated in simple behavioral strategies (e.g., prompt and rewards) to support gains in language and socialization and reduce repetitive behaviors. - Cognitive, academic, and prevocational skills were also addressed. - Parents encouraged to give intervention daily at home. - At each follow-up visit, improvements were noted and recorded as “emerging” or “attained.” - Parents were also advised to place the child in a playschool to improve the group based stimulation of various target symptom clusters.   **Method of Delivery:**   - Each clinical session took about 15–20 minutes. - Intervention strategies were demonstrated to parents, and they were advised to practice at home. - A speech therapist gave the parents15 to 20-minutelong one-on-one sessions and targeted one or two language skills per visit for teaching at home. |
| **Juneja, Mukherjee et al. (2012)**  (ASD)  India | **Structure:**   - Individualized program designed to improve child attention, communication, social skills and behavior - Principles of the Naturalistic method/Milieu method used to create situations during the daily activities of the family to develop joint attention behaviors, such as index pointing, gaze switching, showing, and holding out objects. - Play-based activities to develop joint attention behaviors, social skills, and communication include ball play, toy cars, clay, colored stones, balloons, water play, painting and coloring - Principles of Lovaas’ Applied Behavior Analysis used to improvecommunicationwith gestures and words   **Method of Delivery:**   - A specialist demonstrated methods to parents - Parents acted as therapist - Parents expected to spend 45–90 minutes daily in one to-one setting with child. |
| **Divan,Vajaratkar et al. (2019)**  (ASD)  India | **Structure of the intervention**   - A naturalistic approach for scaffolding and developing communication skills - Used parent-mediated Intervention for Autism Spectrum Disorder in south Asia (PASS) - In the intervention group, PASS Plus was implemented along with treatment as usual.   **Method of Delivery:**   - Parent-child dyad sessions - Initial visit of PASS facilitator included making a 10-minute video of the parent-child play session and using it to explain the behaviours of the child to their parent. - Parents asked to choose strategies to try at home for about 1 hour - New Plus modules introduced in the 4th session, delivered by manualised clinical decision algorithmic approach and common comorbidities are addressed by psychosocial approach - Parents requested to practice personalised communication strategies provided to them for 30 min every day in the intervening fortnight |
| **Padmanabha, Singhi et al., (2019)**  (ASD)  India | **Structure: Parallel group treatment**   - Standard Therapy (ST) group: Intervention included only Standard Therapy, the institution-based standard care for ASD - Sensory Intervention (SI) group: Intervention included predesigned structured Home-Based Sensory Interventions (HSBI) and also Standard Therapy   **Activity sequence of HSBI:**   - Tactile activities - Vestibular Visual Stimulation - Auditory Stimulation   **Method of Delivery**   - SI group: Parent children-dyads ST group: group setting - Parents were given hands on training on HSBI along with a training manual and videos. - For HSBI, children performed simple and easy activities using home-based items (blankets, swing, sofa, bed, wooden horse, dough, rice and soft toys) and a sensory kit (sensory brushes, wool, jute, various sandpapers, flash cards, lighting calls, toys and music CD’s). - HSBI were 45 to 60-minute sessions performed 5 days a week. - Follow up during 2, 4, 8 and 12 week by checking activity logs kept by parents, weekly telephonic reinforcement and review of video tapes of weekly activities. - ST included speech and language services, behaviour modification and applied behaviour analysis. Only ST was provided in Group setting. |
| **Manohar et al. (2019)**  (ASD)  India | **Structure:**  **Intervention group:**   - Outpatient-based delivery intervention included 4 components: joint attention, verbal and motor imitation, social engagement and adaptive skill training. Components based on Naturistic Developmental Behavioural Interventions (NDBI) which involve developmentally appropriate and prerequisite skills in a naturalistic environment using family friendly behavioural strategies, and a strong emphasis on home-based parent mediated component. - Visit 1 through 3: Parental education, pre-intervention assessment and parental training. - Visit 3 through 5: Follow-up, ongoing intervention and ongoing support. - This group also received the treatment as usual (TAU).   **Active Control Group:**   - Involved Treatment as Usual (TAU) - Setting was hospital of regular institutional treatment for ASD   **Method of Delivery:**  **Intervention group:**   - One-on-one flexible content of sessions, overlap was allowed, tailored to the parent’s current level of understanding of the disorder, parental stress and coping, mastery of techniques and needs of individual participants. - Follow up at 4, 8 and 12 weeks - Intervention conducted for short period of time and scheduled during routine outpatient visits. - A total of 5 visits required.   **Active Control group**:   - Monthly visits to the treating doctors. - If patient required, speech and language intervention, occupational therapy and pharmacological intervention for co-morbidities, these were delivered. |

TAU:Involved Treatment as Usual;NDBI: Naturistic Developmental Behavioural Interventions ; ST: Standard Therapy; SI: Sensory Intervention

**S2.2 Table:** Description of intervention programs for the children with ID

| **Study(year)**  **(Type of NDD) and Country** | **Structure of Intervention program** |
| --- | --- |
| **Lakhan (2014)**  (ID)  India | **Structure:**   - One-week parent training on management of behaviour problems - Parents encouraged to practice at home - Behavioral techniques included: (a) Restructuring the environment  (b) Extinction  (c) Token economy  (d) Over correction  (e) Response cost  (f) Differential reinforcement for incompatible/alternate behavior  (g) Differential reinforcement for low‑frequency behavior  (h) Differential reinforcement for other  (i) Physical restraining  (j) Time out   **Method of Delivery:**   - Behavior modification techniques shown to parents - Children received 1‑hour sessions once or twice a month by CBR workers and at least one 1‑hour session by a therapist - Intervention took place at home or in a camp setting, supervised and monitored with parents, non-formal education (NFE) centers, teachers and CBR workers. - Interventions were regularly monitored by the CBR program staff and reviewed annually. |
| **Kurani, Nerurka et al. (2009)**  (ID)  India | **Structure:**   - Included structured classroom programmes, a structured outdoor programme and an unstructured outdoor programme to maintain ‘learnt skills’ (i.e., develop fluency, consistency and generalization) - Interventions included: - Sensory integration, oral-motor therapy to control drooling - Speech and language stimulation - Neurodevelopmental therapy for children with ID - Locomotor and sensory motor training - Behavior modification - Medical intervention for children with comorbid epilepsy and/or dystonia - Therapists were firm but fair in making sure child carried out directives. - Only tasks a child can complete, but which had an element of challenge, were introduced - Support weaned gradually, with authority delegated to more than one caregiver, all of whom were clear on designated behavioral interventions to achieve ‘generalized patterns’ of behaviors   **Method of Delivery:**   - Counselled and trained parents via discussions, family meetings, home visits, referrals to doctors and other paramedical professionals - Used problem solving approaches in one-to-one, small groups and large group follow-up sessions - Classroom sessions with the parent, child and special educator consisted of 2 to 3 hours per day over 5 days - Parents asked to apply training at home |
| **Russell, John et al. (2004)**  (ID)  India | **Structure:**   - Children received an individualized training plan based on their adaptive behavior deficits - Domains of focus included self-care, home living, independent living skills, social skills, sensorimotor skills, language, concepts and control of problem behaviors - Special education and behavioral techniques were used. - Behavioral techniques included chaining, shaping and graded task assignments with either positive or differential reinforcement for each adaptive behaviour deficit   **Method of Delivery:**   - Classroom sessions for adaptive behavior consisted of five 3-hour sessions a week. - Homework assignments emphasized social activities and practicing of the newly learnt adaptive behaviors in daily life. - Parents in theintervention group attended Interactive Group Psycho-education (IGP) for 10 weeks. - Parents of the control group attended didactic lectures as an intervention to enhance parental attitude. - IGP was in closed group sessions, conducted twice a week by psychologists and special educators. - Each 1-hour session focused on aspects of child rearing, developmental milestones and delays, common causes of ID, co-morbidities, skill deficits, problem behaviors, behavioral techniques, sexuality and marriage, as well as legal and social support systems for the intellectually disabled. - Each IGP session alternated between information dissemination, discussion among parents and with therapist and problem solving tasks. - Control group received information on the same topics as didactic lectures without discussions. |
| **Russell, al John et al. (1999)**  (ID)  India | **Structure:**   - IGP focused on child-rearing, developmental milestones and delays, common causes of ID, co-morbidities, skills deficits, problem behaviors, behavioral techniques, sexuality and marriage, and legal and social support systems for individuals with ID in India. - Children attending the therapy programme in both groups were given training in self-care, social skills, pre-vocational skills, and control of problem behaviorsthroughspecial education and behavioral techniques.   **Method of Delivery:**   - IGP was in closed group sessions, conducted twice a week for 10 weeks by psychologists and special educators - Each session alternated between information dissemination, discussion among parents and with therapist and problem-solving tasks. - For control group, classes were conducted for the same period as a didactic lecture, without discussion. |
| **Mohsin and ud Din (2015)**  (ID)  Pakistan | **Structure:**   - Content included: Introduction to ID, introduction to functional skills, use of diaries for identification of functional tasks, hands on activities to complete diaries, and different teaching techniques and reinforcers   **Method of Delivery:**   - Two day training conducted for all parents - Parents and children participated in their homes and were visited by field assistants weekly - Parents selected up to five goals for their children to learn. The program tasks were explained, demonstrated, and role played in the child’s presence. - A Weekly Evolution Report (WER) was completed in the presence of the parents and arrangements were made to deliver a specially developed teaching idea at the next home visit. - Parents shared their difficulties, success stories and other issues at each home visit. |
| **Narayanan, Girimaji et al. (1988)**  (ID)  India | **Structure:**   - Parents and key trainers were counseled intensively about the nature, causes, and associated conditions of ID. - Misconceptions, if any, were clarified. - Special attention paid to emotional problems and motivation for training. - Parents trained on sensory-motor stimulation, development of motor, language and self-help skills, behavior modification techniques, and physiotherapy.   **Method of Delivery:**   - Training through instruction, discussion, demonstration, and in-vivo feed-back - Parents supervise team members. - Targets set for each child at the outset in the areas of gross and fine motor, adaptive, social and language skills. Focus more on parents learning general principles while achieving a particular goal, so newly acquired skills will generalize to home setting - Up to 4 hours per day of professional time by one or more members of the team |

ID: Intellectual Disability; WER: Weekly Evolution Report;IGP: Interactive Group Psycho-education

**S2.3 Table:** Description of intervention programs for the children with ADHD

| **Study(year)(Type of NDD) and Country** | **Structure of Intervention program** |
| --- | --- |
| **Malik, Rooney et al. (2017)**  (ADHD)  India | **Structure:**  Parent training manual, adapted from *Defiant Children* (Barkley, 1997) included:   - Review of information on ADHD: An overview of the nature, developmental course, prognosis and etiology of ADHD - Why children misbehave? Causes of defiant behavior in terms of child characteristics, parent characteristics, situational consequences, parenting style, and stressful family events - Pay attention. Parents trained in more effective ways to attend to child’s behavior to enhance the value of their attention - Attend to child compliance and independent play - Establish home token economy. Parents asked to set up a home token economy to provide external reinforces for activities not intrinsically motivating, such as home chores. - Implement time out for non-compliance - Extend time out to additional non-compliant behaviors - Manage non-compliance in public places. Parents are taught to extrapolate their home management methods to troublesome public places, such as stores, church and restaurants - Improve child school behavior from home   Supporting visual educational package provides parents with comprehensive information on ADHD and introduces basic behavior management techniques.  Visual package including3DVDs featuring the developer of the Defiant Child (Barkley, 2007) were translated and dubbed in Urdu. 1) ADHD: What Do We Know?  2) ADHD: What Can We Do?  3) Managing the Defiant Child: A Guide to Parent Training (Barkley, 2006a, 2006b, 2006c)  **Method of Delivery:**   - Training conducted ingroup sessions - Visual package provided to parents to watch at home at beginning of training - Portions of package utilized during group sessions with real-life examples of challenges families often encounter |
| **Malik and Tariq (2014)**  (ADHD)  Pakistan | **Structure:**   - Same as Malik, Rooney et al. 2017   **Method of Delivery:**   - Same as Malik, Rooney et al. 2017 |
| **Rejani, Oommen et al. (2012)**  (ADHD)  India | **Structure:**   - Parent training conducted using the manual: Defiant children-A clinician’s manual for parent training (Barkley, 1997) - This ten step program consists of explanation of causes of ADHD, teaching principles of behavioral management, and tasks for enhancing parental attending skills, establishing home token systems, using response cost, improving school behavior, managing child’s behavior in public places and handling future behavior problems. - **Attention enhancement training:** Package included coloring, grain sorting, clay modeling, mazes, beading and matching figures and was based on empirical evidence for improving attention deficit and impulsivity. - **Medication management:** Medication used was methylphenidate (MPH) or clonidine as decided by the Psychiatry Consultant. The frequency of consultation for monitoring medication was similar for both groups.   **Method of Delivery:**   - Dosage and side effects were monitored by the senior residents of the Child and Adolescent Mental Health Unit at the end of 2nd, 4th, and 10th week and on follow-ups. - All tasks arranged on the level of difficulty - Tasks introduced in weekly sessions - Parent training spread over ten weekly sessions of one-hour duration each |
| Shah,Chakrabarti  **et al. (2019)**  **(ADHD)**  **India** | **Structure:**   - Multi-point videoconferencing for providing group intervention - Incorporating culturally relevant methods and content, the intervention was designed to improve awareness, parent-child relations, family environment and specific skill-building to handle consequences of ADHD effectively and confidently.   **Method of Delivery:**   - Group parent training sessions of 90 minutes each - Sessions were delivered by video communication through Zoom - Weekly reminders regarding homework and upcoming sessions were also provided by a messaging group online - Face-to-Face intervention done traditionally, with patents visiting the hospital once in 4-8 weeks. |

ADHD: Attention Deficit Hyperactivity Disorder;

**S2.4 Table:** Description of intervention programs for the children with CP

| **Maiya, Shetty et al. (2015)**  (CP)  India | **Structure:**   - Randomly assigned to four groups of 16 children each as follows: Group 1: Manual toothbrush with fluoridated toothpaste, Group 2: Manual toothbrush with fluoridated toothpaste and CHX spray,   Group 3: Powered toothbrush with fluoridated toothpaste, Group 4: Powered toothbrush with fluoridated toothpaste and CHX spray   - Parents andcaregivers applied the custom-made 0.2% CHX spray formulations, one each on the buccal and the lingual surfaces of each arch on a twice-daily basis ½ hour after tooth brushing.   **Method of Delivery:**   - The parents/caregivers/institution staff were first administered a health education program, including the importance of oral hygiene maintenance and knowledge regarding the four preventive home care measures. - A toothbrush (manual or powered as per the group), fluoridated toothpaste and a custom-made CHX spray were provided to the parents/caregivers. Horizontal tooth brushing with manual toothbrush and the correct usage of powered toothbrush with pea-sized amount of fluoridated toothpaste was advised to be performed twice daily by the parents/caregivers. The caregivers were requested to complete 2 minutes of brushing. - Preventive home care measures were advised to be followed for a period of 6 weeks. |
| --- | --- |
| **Arora, Aggarwal et al. (2014)**  (CP)  India | **Structure:**   - An educational film about the etiology, development, management, and role of parents in managing CP - Emphasized that a child with CP can be rehabilitated to perform activities of daily living - Explained role of parental involvement in training the child   **Method of Delivery:**   - Parental knowledge regarding CP was assessed by questionnaire - Following that, subjects were shown educational film. - Follow up visit after one week in which same questionnaire and few additional questions on the film was given |
| [**Karande, Patil et al. (2008**](#_ENREF_4)**)**  (CP)  India | **Structure:**   - A questionnaire and an educational program (either in English, Marathi or Hindi) - Program provided education about the “core basic issues” fop.   **Method of Delivery:**   - Diagnosis of CP was disclosed to the parent(s) at study initiation and questionnaire was administered by interview. - Each parent received the educational program using “flash cards” providing answers to topics covered in the questionnaire. - Childrenwerereferred for early intervention therapy after the educational program was completed. |
| **McConachie, H., et al.et al. (2000)**  (CP)  Bangladesh | **Structure:**   - **Distance Training Packages.** Two parent training programs, one urban and one rural. The Bangladesh Protibondhi Foundation developed distance training packages to provide advice to parents of children with disabilities. - Pictorial manuals illustrate positions, activities, and simple home-made aids and different manuals cover motor skills, speech and language, and cognitive skills. - **Mother-Child Group.** Practice in daily living skills such as using a cup and developmental activities such as sorting by color - **Health Advice Group.** Discussed child’s health as part of the assessment, and then nutritional advice and vitamin supplements were provided as appropriate.   **Method of Delivery:**   - **Distance Training Packages:** One- to 2-hour session in which suggestions appropriate to the child’s stage of development are practiced with parents before they take the manual home - **Mother-Child Group.** Center-based, regular attendance intervention with mother-child stimulation group offered daily, led by a therapist with extra training in physiotherapy - **Health Advice Group.** No detailed advice on positioning or other techniques. Given a box of simple local toys and books for their children to play with at home, items which were not given to parents in the other groups |

Abbreviations: CP: Cerebral Palsy; CRB: Community Based Rehabilitation, IGP: Interactive Group Psycho-education, WER: Weekly Evaluation Report, CAMH:Child and Adolescent Mental Health
