# Supplementary material for: Parent mediated intervention programmes for children and adolescents with neurodevelopmental disorders in South Asia: A systematic review
Source: PLoS One. 2021 Mar 11;16(3):e0247432. doi: 10.1371/journal.pone.0247432 (PMC7951928; doi:10.1371/journal.pone.0247432)
Supplement: S3 Table — (DOC) [file pone.0247432.s004.doc]

**S3 Table.** Search terms for each database

| Data Base | Search Term |
| --- | --- |
| PsycINFO | parent mediated or parents or parent based) and (Intervention or programs or programme ) and (Bangladesh or Afghanistan or India or Pakistan or Nepal or Srilanka or Maldives or Bhutan or South Asia) and (autism or autism spectrum disorder or attention deficit hyperactivity disorder or ADHD or attention deficit disorder or Cerebral palsy or Mental retardation or Intellectual disability ) |
| Pubmed | (("parents"[MeSH Terms] OR "parents"[All Fields] OR "parent"[All Fields]) AND mediated[All Fields] AND ("methods"[MeSH Terms] OR "methods"[All Fields] OR "intervention"[All Fields]) AND ("child"[MeSH Terms] OR "child"[All Fields] OR "children"[All Fields]) AND ("autism spectrum disorder"[MeSH Terms] OR ("autism"[All Fields] AND "spectrum"[All Fields] AND "disorder"[All Fields]) OR "autism spectrum disorder"[All Fields]) or (“Attention Deficit and Disruptive Behavior Disorders” [MeSH Terms] AND ("India"[MeSH Terms]) or ("Pakistan"[MeSH Terms]) or ("Srilanka"[MeSH Terms])or("Afghanistan"[MeSH Terms] )or ("Bangladesh"[MeSH Terms] ) or ("Nepal"[MeSH Terms] ) or ("parents"[MeSH Terms] OR "parents"[All Fields] OR "parent"[All Fields]) AND mediated[All Fields] AND ("methods"[MeSH Terms] OR "methods"[All Fields] OR "intervention"[All Fields]) AND program[All Fields] AND (OR ("parents"[MeSH Terms] OR "parents"[All Fields] OR "parent"[All Fields]) AND mediated[All Fields] AND ("methods"[MeSH Terms] or ("cerebral palsy"[MeSH Terms] OR ( "Intellectual Disabilities"[MeSH Terms] (OR "methods"[All Fields] OR "intervention"[All Fields]) AND program[All Fields] or AND ("conduct disorder"[MeSH Terms] OR ("conduct"[All Fields] AND "disorder"[All Fields]) OR "conduct disorder"[All Fields] OR ("conduct"[All Fields] AND "disorders"[All Fields]) OR "conduct disorders"[All Fields]) |
| Embase | (autism or autism spectrum disorder or ADHD or attention deficit disorder or ADD or Cerebral palsy or Mental retardation or Intellectual disability) and (parents or parent or mothers or fathers) or (mediated or programs or program) and (Bangladesh or Afghanistan or India or Pakistan or Nepal or Srilanka or Maldives or Bhutan or South Asia) |
| Web of Science | (autism or autism spectrum disorder or ADHD or Cerebral palsy or Mental retardation or Intellectual disability) and (parents or parent or mothers or fathers) or ( mediated or programs or program) and (Bangladesh or Afghanistan or India or Pakistan or Nepal or Srilanka or Maldives or Bhutan or South Asia) |
